# Touching beliefs: Using touchscreen technology to elicit subjective expectations in survey research

**DOI:** 10.1371/journal.pone.0207484

**Published:** 2018-11-20

**Authors:** Elisa M. Maffioli, Manoj Mohanan

**Affiliations:** 1 Department of Health Management and Policy, University of Michigan School of Public Health, Ann Arbor, MI, United States of America; 2 Sanford School of Public Policy, Duke University, Durham, NC, United States of America; Northeastern University, CHINA

## Abstract

When making decisions under uncertainty, individuals may form subjective expectations about probabilities of events relevant for their choice. Accurate measurement of subjective expectations is critical for high-quality data needed to analyze individual behavior. This paper reports the development and validity of a new method of eliciting point subjective expectations in developing countries. We developed a touchscreen-based application that combines an animated slider along with dynamic images that change relative sizes based on the probability indicated by the respondent. We compare our method to the more traditional approach of using beans as visual aids. First, we find that respondents have a sound understanding of basic concepts of probability. Second, we test for equality of the distributions elicited with the different methods and find them highly comparable. Third, we provide evidence that respondents report a more favorable opinion about the slider method and more willingness to complete long surveys using the slider rather than beans. Our findings suggest that the slider could be a viable elicitation method for empirical researchers who aim to collect data on subjective expectations in developing countries.

## Ethics statement

Participation was voluntary and verbal informed consent was obtained from each of the respondents. The study protocol was approved by the Duke University Institutional Review Board (Durham, NC) and by Morsel Research and Development Pvt Ltd (Uttar Pradesh, India).

## 1 Introduction

Many of the choices that people make involve considerable uncertainty, because individuals do not have perfect information on the riskiness and possible outcome from a particular action. Then, under uncertainty, individuals rely not only on preferences, but also on their own expectations about probabilities of outcomes relevant for their choice. As a result, it is critical to incorporate in empirical research information on expectations that seeks to understand preferences which lead to observed choices. Omitting such information could lead to problems of identification, since observed decisions might result from a wide range of combination of preferences and expectations [1–2].

Eliciting accurate data on expectations from consumers, however, remains a challenge [2]. While it is possible to directly elicit information on probabilistic expectations from respondents in developed countries [1, 3], this task is far more challenging in developing country contexts where literacy levels are low. With growing attention to the role of subjective expectations in development economics research, empirical researchers have been developing innovative methods to elicit expectations that are easy to implement in survey settings in developing countries [4–9]. Many researchers currently use beans or other visual aids that show scales of outcome responses to elicit subjective expectations [6, 9–13]. The ubiquitous use of smart phones and hand held devices in the developing world presents a unique opportunity to develop new elicitation methods that incorporate technology to enable collection of high quality expectations data.

In this paper, we describe the validation of a new touchscreen-based slider method that we developed to elicit subjective expectations data in rural Uttar Pradesh, India. The touchscreen slider method (slider method, hereafter) consists of eliciting 100-point subjective expectations with the help of an interactive slider application on an Android touchscreen device. There are two key features of the slider application: (1) The respondent moves the slider using a touchscreen interface to indicate the probability that she thinks an event will occur, from 0% at the extreme left, to 100% at the extreme right. (2) The application features dynamic images that change relative sizes based on the probability indicated by the respondent. For example, at 50% probability, the size of images on both extremes is identical, and moving the slider to either end increases the size of the image at that end, while the image on the opposite end shrinks. This approach allows the respondent to be as precise as she would like to be in indicating her subjective expectations in an intuitive manner.

Firstly, we build on a growing literature about the feasibility of collecting subjective expectations data, especially in developing countries, focusing on the methodology adopted to gather high-quality data [2, 4–9]. A variety of methods have traditionally been used to elicit subjective expectations in contexts such as health, education, agricultural production, income and wealth [4, 7–9, 13–17]. First, Likert scaling has been used in surveys to collect data about perceptions of future events occurring. However, the main concern with Likert scaling is that it is very difficult to make interpersonal comparisons, as different respondents may interpret the scale differently.

A second approach consists of asking about probabilities without the use of visual aids, for instance by asking individuals directly about the percent chance that a certain event will happen. However, this method assumes that respondents are educated and understand concepts of probability well enough to articulate their responses in “percent chance”. In developing countries, where many respondents have a low level of education or are illiterate, and the notion of probability is not very commonly used, visual aids were found to be important in explaining this abstract concept [6, 10–13].

Thus, a third method that is now commonly employed in developing countries, involves asking respondents to allocate a given set of stones, balls, beans, or sticks into a number of bins to indicate the probability that a certain event happens. A recent study [6] tested three aspects of this elicitation methodology: the number of beans, the design of the support (predetermined with many intervals or self-anchored with few intervals) and, in the case of self-anchored with few intervals, the ordering of questions about the asked minimum and maximum values. Even though the variations in the design have advantages and disadvantages, the data collected are shown to be robust to the different measurements, and the researchers conclude that the use of 20 beans together with a predetermined support with many intervals is the method that provides more accuracy.

Our paper contributes to this literature by developing a novel point expectations elicitation method and demonstrates its feasibility of eliciting measures of subjective expectations in a developing country rural setting with very low literacy rates. Building on [9] which used a ruler marked from 0 to 100 to elicit point expectations, our slider method makes a twofold improvement. First, the slider, commonly used in web-surveys, is combined with dynamic images which change relative size to help the respondents to better understand the probability indicated. Despite not being able to separate out the effect of the slider by itself and the dynamic images, we show that this method can be implemented on touchscreen based devices and embedded in household surveys collected through tablet or mobile-phone devices. Second, and more importantly, this study benchmarks the touchscreen slider method against the more traditional method of using a discrete number of beans, commonly implemented in surveys in developing countries, to determine whether there are major differences with the use of beans. Specifically, following [6], we use 20 beans as the reference method to elicit probabilities. We administer questions to elicit information on expectations using 20 beans as well as the slider method, randomizing individuals to receive one of the methods. Our study does not aim at saying whether the slider is better (or worst) than the use of 20 beans to elicit subjective expectations in developing countries. Instead, we aim at providing researchers with an alternative method to elicit more precise measures of subjective expectations in low-literacy settings, embedding data collection in commonly used household surveys through touchscreen based devices.

Secondly, we contribute directly to survey literature about slider methods used in web surveys [18–19], where authors compare web sliders to other survey methods. Web sliders are defined either as slider scales [20] or visual analogue scales (VAS) [21]. These two very similar approaches consist of an horizontal line with verbal anchors or visual aids on the far left and right of the bar. However, while respondents have to position a mark on the line to respond with VAS scales (with a discrete or continuous score), the slider scale is formed by a line with a handle that respondents have to move to provide a response [22].

A study [18] compares visual analog scales (VAS) with radio button scales and numeric input scales, concluding that there are not major differences in the response distributions of the slider and alternative approaches. Still, the slider does not have main advantages, instead had higher rates of missing data and longer completion times. Another study [19] instead experimentally compares slider scales and radio buttons scales in horizontal and vertical orientations. The authors find that slider scales were more problematic for participants with less than average education, advising against the use of slider scale and advocating for the use of simpler methods of web data collection. In addition, a more recent study [22] provides evidence that while VAS and slider scales look similar, the use of “point and click” for VAS and “sliding the handle” for the slider have different implications for the data collected. Despite using only three, five, or seven point scales, the authors recommend the use of point scales for discrete variables, while the use of VAS for continuous variables. They authors advice not to use slider scales in web-surveys because of negative effects on data quality.

Our study relates to the slider method used in the experiments above mentioned. We build on this strand of the literature, developing a novel variation of the slider method and applying it to a developing country setting. We use a touchscreen device (tablet), which does not require the handling of a mouse as in web surveys, embedding the subjective expectations data in a household survey. Specifically, we implement a slider scale, which indicates 0 to 100% probability. We also use dynamic images which allow respondents with low-literacy to answer subjective expectations questions. Given the nature of the setting, our slider method is more appropriately compared to the most common method used in developing countries, i.e. the use of 20 beans as visual aids. Learning from this comparison will provide researchers with an application of the slider method in a different setting than in web-surveys. Contrary to past studies which use slider methods in web-surveys in developed contexts [18, 19, 22], we show that this novel slider method is a feasible tool to collect high-quality subjective expectations in a developing country.

Results show that respondents in our sample, despite low levels of education, understand the concept of probability well. They demonstrate a clear and intuitive understanding of nested outcomes and what perfect certainty means at 0% and 100% probability values. We find that distributions of responses elicited through the slider and the beans method, across groups randomized to the two methods of elicitation, are highly comparable. The slider method shows a small, but significant, reduction in the share of responses that are at focal points (such as 50%). Importantly, we also find that respondents favor the slider method over the beans: individuals using the slider method report a lower level of difficulty relative to those using beans (on a scale from 0 to 10, 2.27 vs 3.37, pvalue<0.01). Further, respondents who were interviewed with the slider application said they were less likely to refuse future participation than those using the beans method (on a scale from 0 to 10, 3.40 vs 5.29, pvalue<0.01).

Given the higher precision of the collected data (mainly due to the elicitation of 100-point probability distribution) and the advantages of electronic data collection (eliminate data entry errors, ease of incorporating expectations modules into electronic household surveys, easier monitoring, and interactive touchscreens keeping survey respondents more engaged), we believe the slider method has the potential to become an alternative method to collect subjective expectations data in large household surveys in developing countries.

## 2 Study design

### 2.1 The slider method

**Fig 1:**
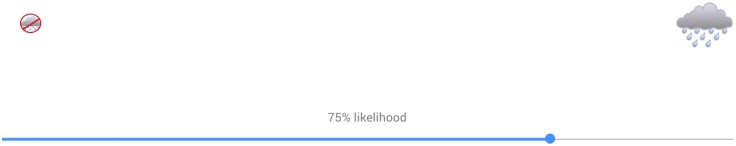
Example of touchscreen application (slider method). The Figure represents the slider method when asking the respondents about the likelihood that it will rain tomorrow.

We developed and validated our touchscreen slider in rural Uttar Pradesh, India, as part of an ongoing study that examines individual decision making for testing and treatment of diabetes. In order to measure expectations in this setting with low levels of literacy, we developed a new touchscreen-based slider method designed to elicit subjective expectations through an Android device. Fig 1 reports an example of the touchscreen user interface, where we ask the respondents about the likelihood that it will rain tomorrow (see S1 Appendix, Fig A for details).

The respondent can indicate the probability that she thinks the event (in the example in Fig 1, rain tomorrow) will occur, from 0% at the extreme left, to 100% at the extreme right, by moving the position of the slider. In addition to the dynamic images that change relative sizes based on the probability indicated by the respondent, the position of the slider also shows the corresponding probability in numbers. Given the mixed evidence on which is the best starting point not to bias the respondents’ answers [23], in our setting we decided to use the far left (0% probability) as default option. However, we programmed the touchscreen-based application as requiring the respondent to touch the slider—even if her intended answer was 0%—in order to proceed to the next question. Hence, whatever default option we chose, the required handle of the slider would not allow respondents to use the default value (0% probability in this case) as their answers.

The visual aids in the slider serve two key purposes. First, the position on the slider yields a response that is internally consistent to framing issues such as a question about the probability of an event occurring *(p)* or the event not occurring *(1-p)*. This is a frequently noted concern in elicitation of probability where respondents’ assessment of *p* and *1-p* do not necessarily add up to 1. In other words, even if the respondent does not explicitly recognize the equivalence of *p* and *1-p*, by indicating a position on the slider the respondent is forced to recognize that stating that an event will occur with probability *p* is the equivalent of stating that it will *not* occur with probability *1-p*, providing an internally consistent answer. Second, the dynamic images with relative sizes combined with the corresponding numerical probability value helps the respondent to better understand and consider the answer she is selecting. For example, as shown in Fig 1, at 75% probability, the position of the slider also displays the number “75%” and the image on the right is three times the size of the image on the left. This approach allows the respondent to be as precise as she would like to be in indicating her subjective expectations. Further, we also trained our enumerators to read the indicated response aloud to the respondent to confirm their answer.

Note that we use the example of the rain (Fig 1) to explain the method to the respondents, we use specific images for few of the initial questions to make the respondent familiar with the method (for example probability of going to the market in 2 days), but we ask the entire set of questions in the survey with standard and consistent visual aids through the use of red bar graphs for the subjective expectations questions related to chronic diseases (see S1 Appendix, Fig B for details).

### 2.2 Study design and analysis

Our validation study was designed to compare subjective expectations data collected using the slider method with data from the beans method, which was developed and used in contexts of low literacy and numeracy to elicit probabilistic expectations. This latter elicitation technique, implemented for example in [11], asks respondents to allocate up to 10 beans on a plate to express the likelihood that an event will be realized. This method was then revised and tested with the use of 20 beans in [6]. In this project, we follow the latest study, using 20 beans as visual aids for the respondents. Practically, we ask respondents to allocate 20 beans into bins based on their subjective probability of an event will occur, allowing then to express probabilities in units of 0.05.

We employed commonly used methods for equivalence trials to calculate the sample size needed for the experiment. We tested whether the difference in the mean probability of an event happening reported using the slider approach is within 7% of the mean probability of the same event happening reported using the beans approach. We performed a sample size calculation for an equivalence test where we assume type I error probability of 5%, power at 80%, standard deviation at 20% and equivalent limit of 7%. We required 140 individuals per group, and thus a total sample size of at least 280 individuals to detect this difference.

We randomized the assignment of 300 study participants to be administered the survey using either 20 beans or the slider. We compare the distribution of expectations data as well as key moments in the distribution across the two groups. Respondents in both groups were asked an identical set of questions. These include questions relating to probability of events in daily life (such as going to market or a major river drying up) as well as questions about the probability of having diabetes and to be alive in 10 or 20 years under different hypothetical scenarios (see S1 Appendix). We primarily compare responses of groups of respondents using the two different methods, comparing the probability distributions elicited using beans or slider. We perform the Komogorov-Sminorv test and the Mann-Whitney test, two leading non-parametric tests of equality that do not require any specific distributional assumption. We also test the equality of moments (mean, median, mode, standard deviation, and percentiles) of the two probability distributions.

## 3 Sample and data

### 3.1 Survey

We recruited respondents among adult members (above 18 years old) from randomly sampled villages in Sultanpur district in Uttar Pradesh, India. Field workers canvassed households door-to-door in villages in the study area until the necessary sample size is reached. In order to limit any possible learning of the methods used in the survey responses from other individuals close to the respondent, only one adult member per each household was enrolled in the study. During the field team visit, if more adults were eligible and willing to participate, field workers were instructed to enroll the person with the first name, first in alphabetical order. Once enrolled in the study, respondents were randomized in one of the two groups through the application developed on the Android device, and clear instructions about which method to use for each set of questions appeared on the device used by field workers to carry-on interviews. We elicited expectations data from 150 respondents respondents using 20 beans, and from another 150 respondents using the slider, for a total of 300 individuals in the sample from 6 villages.

Basic socio-demographic information about the respondent and her household are captured at the beginning of the survey. A longer set of questions about household characteristics, such as house characteristics and assets, which are combined to have an indicator of household wealth, are placed at the end of the survey to avoid tiring respondents unnecessarily at the beginning of the interview.

Using a set of 6 questions, we seek to learn whether respondents understand the concept of probability. The first question elicits the probability of picking one red ball out of 5 balls (2 red, 3 black). The next two questions test whether respondents know the concept of nested probabilities. We follow [6] and ask about the likelihood of going to the market in the next two days and in the next two weeks. If the question is understood correctly, the likelihood of going to the market in the next two days is expected to be lower than the likelihood of going to the market in the next two weeks. The next two questions ask about an event that is likely to have zero probability (the river Ganga will dry up tomorrow) and a certain event (Diwali day will be a public holiday next year). We finally ask the respondent about the likelihood that a randomly selected student in 10th standard class is a girl. This question provides an estimate from the survey that can be compared against a “true” estimate.

All the other subjective expectations questions asked in the survey are related to the probability of having diabetes, being alive in 10 or 20 years or being alive in 10 or 20 years in the hypothetical scenarios the respondent is found to have or not have diabetes. We used this type of questions because diabetes is a common problem in the study area that everyone is aware of, and provides an excellent example of applied research questions that might be implemented using the slider method. See S1 Appendix, Fig C for a list of questions.

### 3.2 Sample

Most of the respondents (about 77%) are females and on average they are 40 years old (Table 1). More than 50% of the respondents have none (33%) or completed primary education (17%), and 52% work in agriculture. Half of the sample is from low caste (Forward caste, Scheduled Caste, Scheduled Tribes). Most of the households have electricity (76%), and they own assets such as a mobile phone (89%). Summarizing all the information about assets owned and house characteristics of the respondents, we constructed an index of wealth through principal component analysis. We followed the methodology used in the Demographic and Health Surveys [24]: we developed an index that takes values from 1 to 5 and we define wealth quintiles as lowest, second, middle, fourth, and highest. On average households in our sample are in the middle quintile of the wealth distribution. More details on the variables used to construct the index are presented in S1 Appendix, Table A. Table 1 also shows that the random assignment of respondents to use the slider and the beans method yielded balance across socio-demographic characteristics. We do not find any statistically significant differences among the two groups.

**Table 1:** Descriptive statistics and balance check.

	Sample	Beans	Slider	Pvalue	N(Beans/Slider)
Female	0.77	0.76	0.79	0.583	150 / 150
	(0.42)	(0.43)	(0.41)		
Age	40.35	41.10	39.61	0.341	150 / 150
	13.55	(13.13)	(13.97)		
Married	0.88	0.89	0.87	0.600	150 / 150
	(0.33)	(0.32)	(0.34)		
Hindu	0.95	0.93	0.96	0.306	150 / 150
	(0.23)	(0.25)	(0.20)		
Low caste	0.52	0.52	0.53	0.908	150 / 150
	(0.50)	(0.50)	(0.50)		
Low education	0.51	0.53	0.49	0.490	150 / 150
	(0.50)	(0.50)	(0.50)		
Occ agriculture	0.52	0.53	0.51	0.730	150 / 150
	(0.50)	(0.50)	(0.50)		
Own house	0.99	1.00	0.99	0.318	150 / 150
	(0.06)	(0.00)	(0.08)		
Wealth Index (1-5)	2.90	2.99	2.81	0.278	150 / 150
	(1.44)	(1.47)	(1.40)		
Income (30 days)	6993.33	7267.33	6719.33	0.524	150 / 150
	(7425.52)	(9108.93)	(5246.22)		
Food expenditures (30 days)	3612.67	3613.33	3612.00	0.996	150 / 150
	(2319.98)	(2477.35)	(2159.51)		

Notes: This table reports the descriptive statistics and the t test of the difference in means between the respondents who used the beans method and the respondents who used the slider method. Means are reported, with standard deviations in parenthesis. Variables are defined as whether the respondent is female; age of the respondent; whether the respondent is married; whether the respondent is of Hindu religion; whether the respondent belongs to a low caste, such as Forward caste, Scheduled Caste, Scheduled Tribes; whether the respondent has none or completed primary education; whether the respondent’s primary occupation is in agriculture; whether the household owns a house; a wealth index which takes values from 1 to 5 and defines wealth quintiles as lowest, second, middle, fourth, and highest, following Demographic Health Surveys; the household’s income in the past 30 days (in Rupees); the household food expenditure in the past 30 days (in Rupees). * *p* < .10, ** *p* < .05, *** *p* < .01.

### 3.3 Subjective expectations data

Table 2 summarizes responses on the concept of probabilities (Panel A), and whether there are inconsistencies in the responses (Panel B).

**Table 2:** Descriptive statistics, subjective expectations.

	Mean	Sd	Min	Max
Panel A: Subjective expectations				
*a. Test questions*				
Prob pick ball (2/5)	34.52	17.09	0.00	100.00
Prob student is girl (1/2)	46.03	13.60	0.00	100.00
Prob river dries up (0%)	0.00	0.00	0.00	0.00
Prob Diwali holiday (100%)	97.96	13.18	0.00	100.00
Prob going to market in 2 days	69.78	37.17	0.00	100.00
Prob going to market in 2 weeks	83.50	26.57	0.00	100.00
*b. Probabilities to be alive*				
Prob to be alive in 10yrs	87.49	22.41	10.00	100.00
Prob to be alive in 20yrs	74.00	31.08	0.00	100.00
Age to live	71.51	8.63	18.00	90.00
*b1. With diabetes*				
Prob to be alive in 10yrs	47.13	23.11	0.00	100.00
Prob to be alive in 20yrs	25.58	18.59	0.00	100.00
Age to live	45.51	9.45	0.00	70.00
*b2. Without diabetes*				
Prob to be alive in 10yrs	95.14	12.85	22.00	100.00
Prob to be alive in 20yrs	88.14	19.48	15.00	100.00
Age to live	73.49	9.16	20.00	90.00
Panel B: Percentage of inconsistent responses				
Prob river dries up (0%)	0.00	0.00	0.00	0.00
Prob Diwali holiday (100%)	5.00	21.83	0.00	100.00
Prob going to market	9.33	29.14	0.00	100.00
Prob to be alive in 10 years (with/wo diabetes)	1.33	11.49	0.00	100.00
Prob to be alive in 20 years (with/wo diabetes)	1.67	12.82	0.00	100.00

Notes: This table reports summary statistics for the subjective expectations data for the sample of 300 respondents. Only one observation is missing in the probability of being alive in 20 years with and without diabetes. The distribution of subjective expectations elicited through the beans methods (1 to 20 beans) has been converted to the correspondent values from 0% to 100%. In Panel B, “Prob river dries up (0%)” equals 1 if the probability is different than 0%, while “Prob Diwali holiday (100%)” equals 1 if probability is different than 100%; “Prob going to market” equals 1 if the probability of going to market in 2 days is higher than going in two weeks; “Prob to be alive in 10 (20) years (with/without diabetes)” equals 1 if the probability of being alive in 10 (20) years with diabetes is higher than the probability of being alive in 10 (20) years without diabetes.

First, individuals appear to intuitively understand the concept of extreme certainty (0% and 100% probabilities). In fact, 0% of the sample reports inconsistent values for the 0% probability question and only 5% of the sample reports inconsistent values for the 100% probability question, as defined by values that are different from the correct answers of 0% and 100% respectively (Panel B).

Individuals also have a strong understanding of the basic property of probability theory, by respecting the monotonicity of nested events. The likelihood of going to the market in two days is lower than going in two weeks, with only 9% of responses reported being inconsistent. One may wonder why we find this high inconsistency rate, and whether this is due a wrong interpretation of the second question (probability of going to the market in two weeks), based on the answer to the first one (probability of going to the market in one week). In fact, the second question could be interpreted also as: “Other than your trip to the market in the next two days, how likely is that you would go again to the market in the next two weeks?”, leading to a potential lower probability in the second question compared to the first. Compared to other studies (for example [11] which, using the beans method, ask enumerators to leave on the plate the beans expressing the likelihood of going to the market within two days to answer the next question), in our study, we decided not to anchor the second probability question to the first one. However, both in the translation and in the enumerators training, we clarified that the term used for the time-frame “in two weeks” contains “in two days”; in other words, that the likelihood of going to the market in two weeks contains the likelihood of going to the market in two days, and that the two weeks time-frame do not refer to the additional probability of going in two weeks other than the trip to the market tomorrow or the day after tomorrow. Moreover, note that about one third of these inconsistencies have probability values of going to the market in two days or two weeks very close to each other (less than 5 percentage points apart), indicating that the respondents might have wanted to indicate a similar likelihood for going to the market in two weeks as the one in two days.

We also explored similar concepts of nested probability with probability of being alive at 10 and 20 years from the date of survey, in cases with and without diabetes. The probability of being alive at 10 years from the date of survey is on average higher than the probability of being alive at 20 years. Furthermore, respondents believe that their probability of being alive at 10 or 20 years from the date of survey is much lower in the hypothetical scenario individuals have diabetes compared to a scenario in which they do not have diabetes. In these cases, for example, inconsistent responses are present in less than 2% of the sample (1.33% and 1.67% for the probability to be alive in 10 or 20 years respectively). Finally, the objective the probability of finding a girl, picking a random student in a 10th grade class is not far from to the true value (50%).

One additional concern we need to check in our data is related to the possibility of indicating focal points in the responses [25]: the frequent use of 0%, 50% and 100% can hide a lack of understanding of the probabilities. In particular, the use of 50% as common probability might be related to uncertainty in the respondent’s responses [26]. Notice, however, that given our experimental design, the two methods used (beans or slider) can also influence the percentage of focal responses in the data. We present data on how focal points change depending on the method used to elicit responses, with the goal of drawing some conclusions about how frequent focal points are.

**Fig 2:**
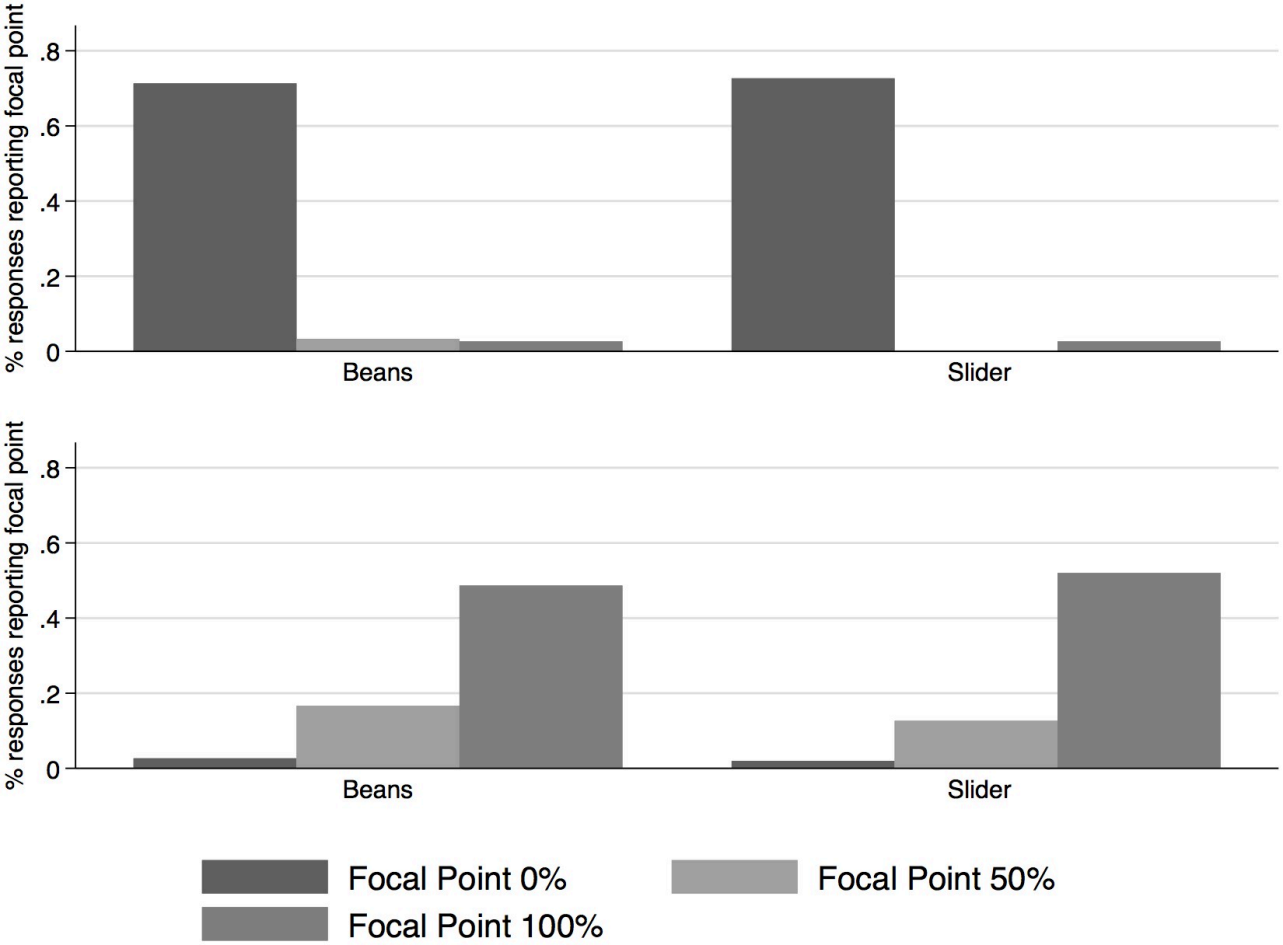
Focal points in probability of having diabetes and being alive in 20 years (beans vs slider). The Figure represents the percent of responses in the probability of having diabetes (top panel) and being alive in 20 years (bottom panel) that are focal points (0%, 50%, 100%), by elicitation method (beans vs slider).

We describe focal points by the use of beans and the use of slider in Fig 2, taking as example the probability of having diabetes and being alive in 20 years. When comparing focal point responses, it is however important to consider that the use of slider might add measurement error in the responses. For instance, an individual who wants to answer 50% with the slider, might end up stopping moving the slider at 49% by mistake. Thus, when we compare all the responses elicited with slider and beans, we re-scale the slider distribution by adding (plus or minus) 2.5 percentage points around each focal point in order to capture potential measurement error of the slider. Practically, to define 0% focal points we include plus 2.5 percentage points, to define 100% focal points we include minus 2.5 percentage points, to define 50% focal points we include plus and minus 2.5 percentage points, to allow some margin of error of the respondents around 50%. This choice of about 2.5 percentage points was discretionary, but note that the results of this analysis are robust to considering slightly smaller or bigger margins of errors or not considering any adjustment around the focal points (not shown). Taking as example the probability of having diabetes (Fig 2, top panel), we do not find statistically significant difference in the percentages of focal responses at 0% and 100% (p-value 0.7979 and 1.0000), but there exists statistically significant difference among focal responses at 50% (p-value 0.0241). Taking as example the probability of being alive in 20 years (Fig 2, bottom panel), we also do not find statistically significant differences (p-values 0.7033, 0.3291, 0.5652 at 0%, 50% and 100% focal points, respectively). Thus, it does seem that there are no major differences among the two methods in term of focal points. If anything, the slider method performs better than beans as far as 50% focal point is concerned.

A final concern might be non-item responses. We do not report any comparisons in non-items responses between slider and beans methods because the data have only one missing value in the probability of being alive in 20 years out of 300 observations.

Another potentially appealing reason to implement a touchscreen slider might be if respondents find this method of elicitation to be easier or less tedious. To fully understand the potential advantages of the slider method, we compare respondents’ opinions on the method they were randomized to. We asked two questions on a scale from 1 to 10 (from very easy to very difficult): (i) If we had to conduct large scale surveys using the slider (bean for those who were in this group) method, how difficult does the respondent think it would be for an average person in rural Uttar Pradesh to using the slider (bean) method; (ii) If our future survey would ask respondents to answer 50 questions using the method the respondent used, how likely is it that an average person in rural Uttar Pradesh might refuse to participate in the survey because of the time it might take and the level of difficulty. We also ask surveyors to judge the accuracy of answers provided by the respondents, and the seriousness and attentiveness of the respondent (on a scale from 1-4 from excellent to very bad) during the survey.

In Table 3 we show comparisons of mean difference among the two groups on perceptions of elicitation methods. Overall, we find that respondents who used the slider had more favorable opinions about the method. Respondents’ difficulty rating for the slider method was almost half a standard deviation less than for the beans method. Respondents randomized to the slider method also reported a much lower (approximately 0.7 SD) probability for respondents refusing to complete a longer survey relative to the ones in the beans group, suggesting that the slider method imposes a lower burden on the respondent. The data confirms that there are no statistically significant differences in the level of attention the respondents used in the two methods, despite the use of the tablet being new to most of the respondents. This suggests that differences in their perceptions about the method used might not be due to differences in how much involved they were in the survey.

**Table 3:** Opinion on the elicitation method used and respondent’s involvement in the survey.

	Beans	Slider	Pvalue	N(Beans/Slider)
Method is difficult (0-10)	3.37	2.27	0.000***	150 / 150
	(2.27)	(2.49)		
Method likely to refuse (0-10)	5.29	3.40	0.000***	150 / 150
	(2.89)	(2.47)		
Resp is not accurate (1-4)	1.76	1.82	0.451	150 / 150
	(0.68)	(0.70)		
Resp is not attentive (1-4)	2.13	2.11	0.890	150 / 150
	(0.80)	(0.87)		

Notes: The table reports the t test of the difference in means between the respondents who used the slider or the beans methods. Means are reported, with standard deviations in parenthesis.

* *p* < .10,

** *p* < .05,

*** *p* < .01.

The variables are constructed from the following survey questions: on a scale from 1 to 10 (i) if we had to conduct large scale surveys using the slider (bean) method, how difficult do you think would be for an average person in rural Uttar Pradesh to use the slider (bean) method?, and (ii) if our future survey would ask respondents to answer 50 questions using the slider (bean) method you used, how likely is it that an average person in rural Uttar Pradesh might refuse to participate in the survey because of the time it might take and the level of difficulty?. We also ask surveyors on a scale from 1 to 4 (from excellent to very bad), (iii) what is your evaluation of the accuracy of answers provided by the respondents, and, (iv) what is your evaluation of the seriousness and attentiveness of the respondent during the survey.

Finally we also collected data on time required for implementing the survey. Our survey, in its entirety, lasted on average 32 minutes. However, we do not find any statistically significant difference between the survey time of respondents using the beans method (32.2 minutes) and the slider method (31.9 minutes).

Overall, we can conclude that respondents in the sample, despite low levels of education, understand the concepts of probability including events with certainty (0% and 100% probabilities), and nested probabilities as well. The slider method, which is more favorably rated by respondents, also has slightly lower percentage of focal responses.

## 4 Results: Comparison of distributions, by method of elicitation

**Fig 3:**
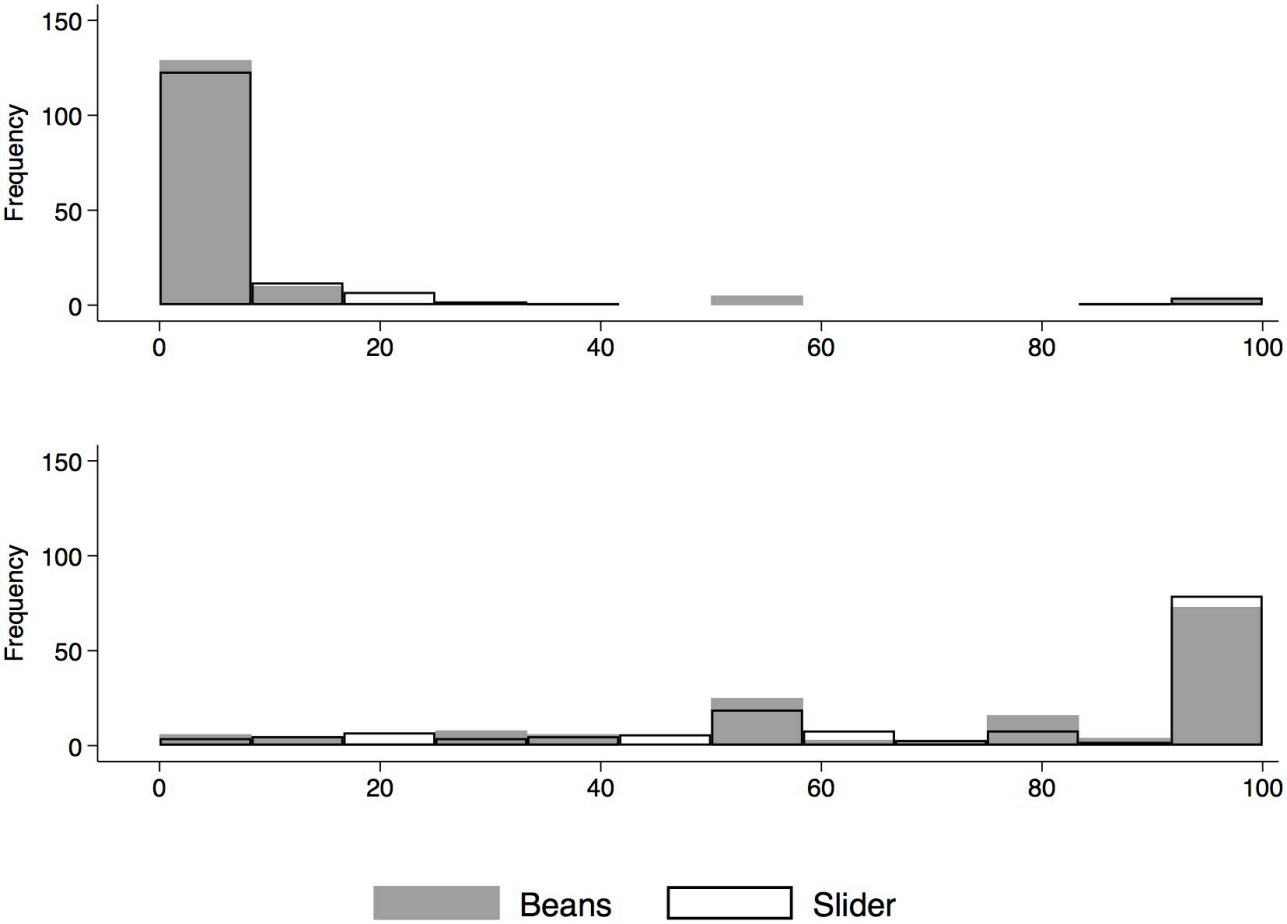
Distribution of probability of having diabetes and being alive in 20 years (beans vs slider). The Figure represents the frequency distributions of the probability of having diabetes (top panel) and being alive in 20 years (bottom panel), by elicitation method (beans vs slider).

We compare differences in distributions of responses elicited with beans and slider across the two groups. In particular, we focus our attention on the subjective probability of having diabetes and being alive in 20 years as examples, but we can implement this exercise with any other response elicited in the survey. Fig 3 shows that distributions of subjective probabilities elicited with beans and slider are highly comparable.

The distributions are presented in Panels A in Table 4, while Panels B describe tests of equality for moments of the distributions and tests for equality of distributions per se. When we formally test the equality of the distributions or moments of the distribution across the two groups, we find that the test for the mean, the standard deviation, the median, and Mann-Whitney test and the Kolmogorov-Smirnov test do not reject the null hypothesis neither in the case of the probability of having diabetes nor of the probability of being alive in 20 years.

**Table 4:** Comparison of distributions of probability of having diabetes and being alive in 20 years (beans vs slider).

**(1) Probability of having diabetes**							
Panel A: Moments of Distribution	mean	sd	p5	p25	p50	p75	p95
Beans	6.03	18.25	0.00	0.00	0.00	5.00	50.00
Slider	6.19	18.27	0.00	0.00	0.00	4.00	25.00
Panel B: P-values for testing equality	t-test	t-test	test	test			
	mean	sd	KS	MW			
P-Value	0.942	0.990	0.934	0.973			
**(2) Probability of being alive in 20 years**							
Panel A: Moments of Distribution	mean	sd	p5	p25	p50	p75	p95
Beans	73.53	31.27	10.00	50.00	90.00	100.00	100.00
Slider	74.47	31.00	14.00	50.00	100.00	100.00	100.00
Panel B: P-values for testing equality	t-test	t-test	test	test			
	mean	sd	KS	MW			
P-Value	0.795	0.915	0.977	0.773			

Notes: This table reports the comparison in the distributions of the subjective expectations elicited through beans or slider methods. Panel A reports the moments of the distributions. Panel B reports the p-values for the test of equality in the distributions: KS and MW denote the Kolmogorov Smirnov test and the Mann Whitney rank-sum test, respectively.

## 5 Conclusion

Collecting subjective expectations data in developing countries, where literacy levels are low, is a big challenge because individuals are not familiar with concepts of probability. However, it is more and more important to be able to collect high-quality expectations data that, combined with individual preferences, help researchers to model population decisions in health, education, and in other contexts. Researchers have tried to measure subjective expectations in rural and developing settings in survey-research through the use of visual aids. The use of physical objects, however, might not be the easiest implementable way to collect these types of data in large household surveys.

We developed and tested a novel method to collect subjective expectations data: an interactive slider application on an Android touchscreen device embedded in the main digital survey. Through this method, the respondent can indicate the probability that she thinks an event will occur, from 0% at the extreme left, to 100% at the extreme right, by moving the position of the slider. In addition to the dynamic images that change relative sizes based on the probability indicated by the respondent, the position of the slider also shows the corresponding probability in numbers. This approach allows the respondent to be far more precise in indicating her subjective expectations relative to the size of bins (5 percentage points with 20 bins). In principle, it is possible to program the touchscreen slider application with a fully continuous set of probability measures with several decimals. However for simplicity and intuitiveness we implemented sliders with a range of 0 to 100% probability, in discrete increments of one percentage point, in practice eliciting subjective expectations in 100-point scale.

The validation of the slider method yields several important findings that are of interest to empirical researchers who aim to collect data on probability and subjective expectations in developing countries. We find that despite low literacy in our settings, respondents have an intuitive understanding of probability, of events with certainty, and nested probabilities. The slider method appears to have several advantages over the beans method. First, the slider yields slightly lower percentage of focal responses. Second—this is probably more important for empirical researchers conducting field surveys—respondents report a more favorable opinion about the slider method and report more willingness to complete long surveys using the slider rather than beans. Furthermore, the slider method also has potential other advantages including eliminating data entry errors, ease of incorporating expectations modules into electronic household surveys, easier monitoring, and interactive touchscreens that keep survey respondents more engaged. Although the slider is an appealing option for elicitation of point estimates of probability, one limitation is that it might not be as suitable for elicitation of distributions. For instance, if the question at hand was not the probability of rain tomorrow, but whether the rainfall is likely to be mild, moderate, or heavy, it is not clear whether the current simple slider might have significant advantages over currently available methods.

While further exploration and validation in other settings is necessary, we believe the slider method has the potential to become a feasible alternative method to collect subjective expectations data in field settings in developing countries.

## Supporting information

S1 Appendix Fig A, The Tool. Fig B, Other Examples. Fig C, The Survey Instrument. Table A, Variables for PCA—Wealth Index.
(PDF)
