# Supplementary material for: Touching beliefs: Using touchscreen technology to elicit subjective expectations in survey research
Source: PLoS One. 2018 Nov 20;13(11):e0207484. doi: 10.1371/journal.pone.0207484 (PMC6245744; doi:10.1371/journal.pone.0207484)
Supplement: S1 Appendix — Fig A, The Tool. Fig B, Other Examples. Fig C, The Survey Instrument. Table A, Variables for PCA—Wealth Index. (PDF) [file pone.0207484.s001.pdf]

Supporting Information

S1 Fig A. The Tool

Rain example

[The following example will help the respondent to understand the concept of probability and how to answer during the survey using the touch-screen tablet. The interviewer reads out the text and then shows the figures on the tablet; the interviewer let the respondent play with the screen to get a sense of the probability.]

I would like to ask you about the chance or likelihood that certain events are going to happen. As you know, there are many events in our lives that are uncertain. For example, we can guess about the chance it will rain tomorrow, but cannot say for sure it will rain on any given day. Also, the chance it might rain will also depend on the season. For example, in this rainy season (July-September), you might expect that there is a high chance that it will rain tomorrow. But there is no guarantee that it will rain for sure. On the other hand, if I had asked you this question about a day in summer (March-June), you might have expected that the chance of rain is very low. But again, completely unexpected rains can arrive even in March! Using this screen, you can express how certain you think these uncertain events might occur (like whether it will rain tomorrow). When the ball is in the middle of the line, it shows you think it is equally likely that it will rain or not rain. As you move the ball towards the rain, you indicate that you are more and more certain it will rain. If you slide it all the way to the right, you are 100% absolutely certain it will rain.

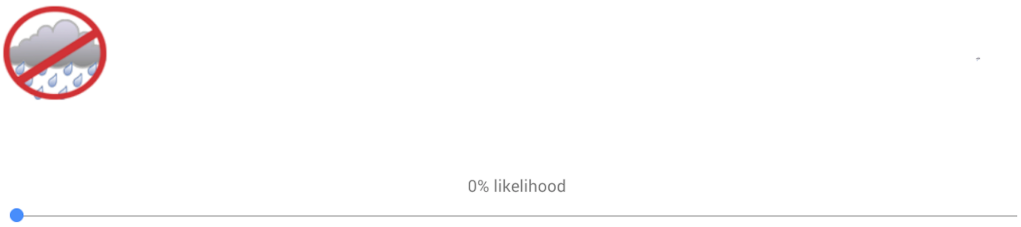

[Example of zero likelihood of rain tomorrow]

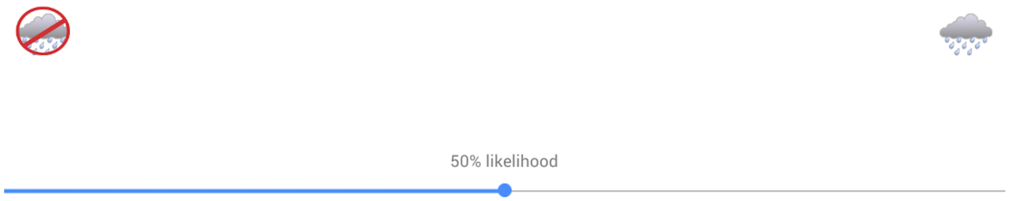

[Example of equal likelihood of rain or no rain]

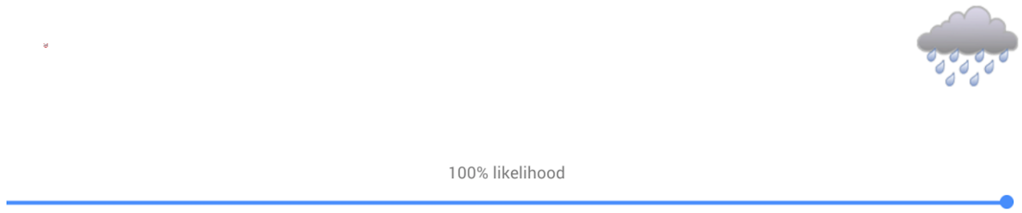

[Example of 100% likelihood of rain]

S1 Fig B. Other Examples

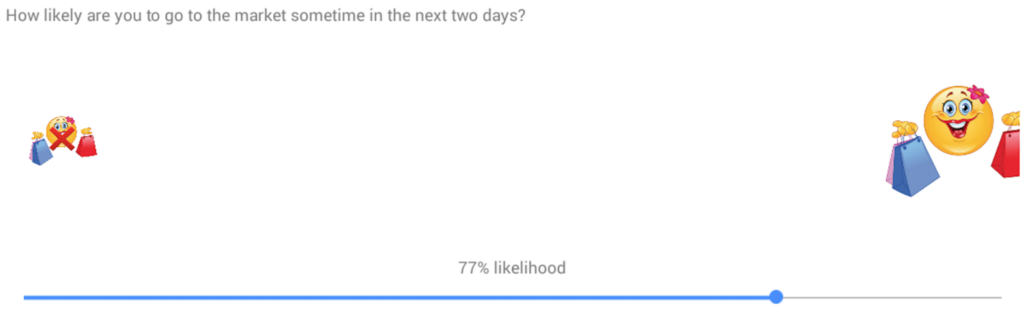

[Example of likelihood of going to the market in two days]

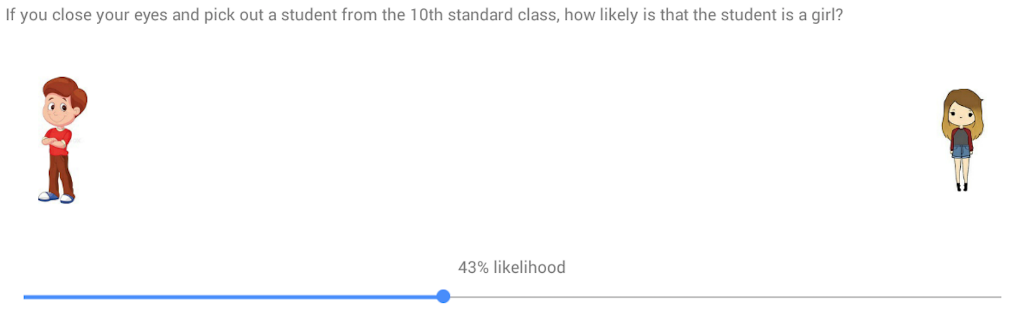

[Example of likelihood of picking a girl student in a class]

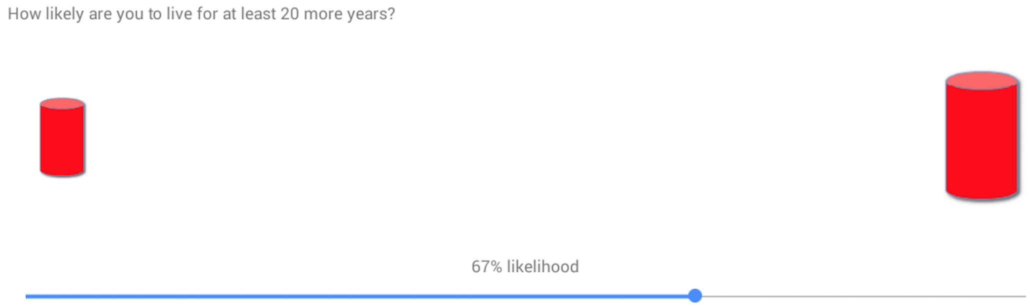

[Example of likelihood of being alive in 20 years]

S1 Fig C. The Survey Instrument

Set of questions 1: concept of probability

1. Imagine you have five balls in a basket: 2 are red, 3 are black. If you have to pick one ball without looking, how is the likelihood that you will be picking a red ball?
2. How likely are you to go to the market sometime in the next two days?
3. How likely are you to go to the market sometime in the next two weeks?
4. How likely is it that the Ganga River will dry up tomorrow?
5. How likely is that Diwali day will be public holiday?
6. If you close your eyes and pick out a student from the 10th standard class, how likely is that the student is a girl?

Set of questions 2: being alive in 10 or 20 years and diabetes

1. How likely are you to live for at least 10 more years?
2. How likely are you to live for at least 20 more years?
3. How old do you believe that you will live to be?

If today an adult person learns that she/he does NOT have DIABETES:

1. How likely are you to live for at least 10 more years?
2. How likely are you to live for at least 20 more years?
3. How old do you believe that you will live to be?

If today an adult person is diagnosed with DIABETES, but she/he NEVER TAKES TREATMENT for the rest of her/his life, and complications will never occur:

1. How likely are you to live for at least 10 more years?
2. How likely are you to live for at least 20 more years?
3. How old do you believe that you will live to be?

How likely is that you have DIABETES?

S1 Table A. Variables for PCA - Wealth Index

	Mean	Sd
Have electricity	0.757	0.430
Own television	0.417	0.494
Own refrigerator	0.0400	0.196
Own mobile phone	0.887	0.318
Own motorcycle	0.330	0.471
Own bank account	0.960	0.196
Improved wall material	0.630	0.484
Improved floor material	0.417	0.494
Improved roof material	0.237	0.426

Notes: This table reports the descriptive statistics of the discrete variables used in the Principal Component Analysis to create the Wealth Index reported in Table 1, for the sample of 300 respondents.
